# Transcriptomic analysis provides a new insight: Oleuropein reverses high glucose-induced osteogenic inhibition in bone marrow mesenchymal stem cells *via* Wnt10b activation

**DOI:** 10.3389/fbioe.2022.990507

**Published:** 2022-08-26

**Authors:** An Lao, Yu Chen, Yiting Sun, Tiange Wang, Kaili Lin, Jiaqiang Liu, Jianyong Wu

**Affiliations:** ^1^ Department of Stomatology, Xin Hua Hospital, Shanghai Jiao Tong University School of Medicine, Shanghai, China; ^2^ Shanghai Key Laboratory of Stomatology, National Center for Stomatology, National Clinical Research Center for Oral Diseases, Department of Oral and Cranio-maxillofacial Surgery, Shanghai Jiao Tong University School of Medicine, College of Stomatology, Shanghai Ninth People’s Hospital, Shanghai Jiao Tong University, Shanghai, China

**Keywords:** transcriptome sequencing, BMSCs, high glucose microenvironment, Wnt10b, oleuropein

## Abstract

Adverse events of diabetes mellitus (DM) include bone damages, such as the increased incidence of osteoporosis and bone fractures, which are known as diabetic osteopathy. The pathogenic mechanism of diabetic osteopathy is complex, and hyperglycemia is a vital cause involved in it. Bone marrow mesenchymal stem cells (BMSCs) exert a significant effect on bone formation. Therefore, in this paper, transcriptomic changes of BMSCs cultured in high glucose (35 mM) for 30 days are mainly investigated. In addition, 794 up-regulated genes and 1,162 down-regulated genes were identified. Then, biological functions of the differentially expressed genes in the high glucose microenvironment were investigated by two kinds of functional analyses. Gene Set Enrichment Analysis was also applied to focus on the significant gene sets and it is found that Wnt10b expression witnessed a remarkable decrease in BMSCs under the high glucose microenvironment. At last, *in vitro* experiments revealed that oleuropein effectively reversed high glucose-induced osteogenic inhibition *via* activating Wnt10b in BMSCs.

## GRAPHICAL ABSTRACT

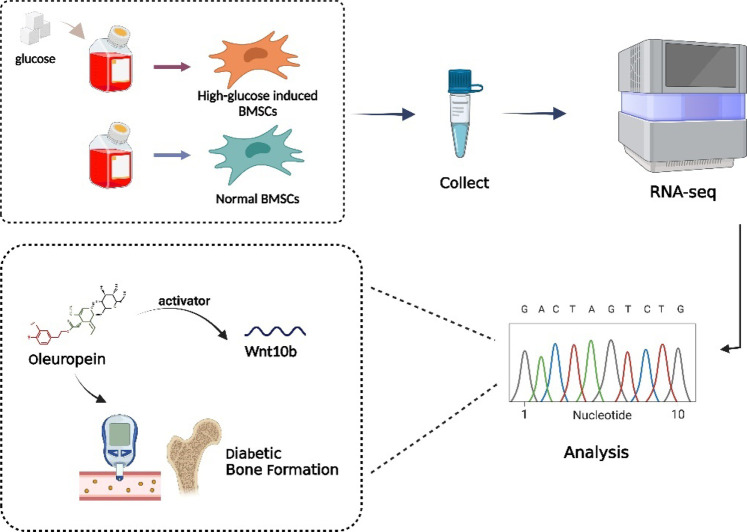

## Highlights

Transcriptomic analysis indicates potential genes or pathways of influencing diabetic bone formation, which also providing a potential medicine—oleuropein. And we proved oleuropein reverses high glucose-induced osteogenic inhibition *via* wnt10b activation.

## Introduction

DM is a common metabolic disease characterized by chronic hyperglycemia (Magliano et al., 2020), with an increasing prevalence worldwide (Zimmet et al., 2016). Its chronic complications exert negative effects on various organs, including bones, resulting in osteoporosis and fragility fractures, which are called “diabetic osteopathy”. With a high morbidity and mortality, diabetic osteopathy is usually associated with high health-care costs (Hamann et al., 2012; Napoli et al., 2017). Diabetic osteopathy has complex underlying mechanisms, and is a result of the interaction of several factors, including a high glucose level and bone metabolism (Epstein et al., 2016; Chen et al., 2022).

Osteoblasts derived from mesenchymal stem cells control bone formation and osteoclasts stemmed from hematopoietic stem cells contribute to bone absorption. These dynamic processes are the key to the balance of bone metabolism (Rachner et al., 2011). If bone resorption and formation are out of balance, metabolic bone diseases such as age-related osteoporosis arise (Tencerova and Kassem, 2016). In the recent years, researchers have paid increasing attention to the biology of bone marrow, which is a chamber of bones and regulates bone homeostasis (Vandoorne et al., 2018; Zhou et al., 2019). Abnormalities of bone marrow microenvironment dynamics can also lead to metabolic bone diseases. Diabetes mellitus can cause various abnormalities of bone marrow microenvironment, the most intuitive of it is high glucose, which may contribute to the skeletal complications.

BMSCs are multipotent stem cells that exist in bone marrow stroma and can differentiate into osteoblasts (Guan et al., 2012; Tencerova and Kassem, 2016; Wei et al., 2022). It has been reported that there are reduced bone mass and inhibited osteogenesis in the condition of diabetes mellitus (Gilbert and Pratley, 2015), suggesting that an abnormal differentiation of BMSCs under high glucose microenvironment may be a possible pathogenetic mechanism, but it remained to be further investigated.

Several researches have been reported to explore the gene transcripts of BMSCs. Ao Zhou et al. (2016) investigated the effects of lipopolysaccharide (LPS) treatment on the changes of whole genome splicing pattern in mouse BMSCs, revealing that splicing patterns of 197 exons were changed by LPS. Serena Rubina Baglio et al. (2015) made small RNA sequencing analysis of exosomes secreted by mesenchymal stem cells of the adult derived from two different sources: adipose and bone marrow. There is a lack of the sequencing analysis of BMSCs in a high glucose microenvironment, which requires further exploration and study.

RNA-seq analysis was carried out in this paper to characterize the transcriptomic changes of BMSCs in the high glucose microenvironment. Besides, whether a high glucose level led to BMSCs dysfunction and how it was regulated by a number of factors were explored. On the basis of RNA-seq results, we found that oleuropein may be the potential molecular compound to reverse high glucose-induced osteogenic inhibition. It may provide new insights and treatment methods into diabetic osteopathy.

## Materials and methods

### Animals and cell culture

4-week-old male C57BL/6 mice were bought from the Laboratory Animal Center of the 9th People’s Hospital, affiliated to Shanghai Jiao Tong University School of Medicine. All the experiments of this study were approved by the Shanghai Jiao Tong University Animal Care and Use Committee. Metaphysis of mice femurs and tibias isolated from adherent soft tissue was cut out, and the marrow was rinsed with 10 ml Minimum Essential Medium Alpha (αMEM) containing 10% fetal bovine serum (FBS) and 1% penicillin and streptomycin (Gibco) and then cultured in a humid environment at 5% CO_2_ and 37°C. On third day, the medium was changed to a new αMEM medium containing 5.5 mM or 35 mM glucose (Sigma-Aldrich, Shanghai, China), and the cells were cultured for another 30 days.

### Flow cytometry

Before exposure to the high glucose microenvironment, flow cytometry was applied to verify the cells were stem cells. Mouse mesenchymal stem cell detection kit (MUXMX-09011, Cyagen, China) was used according to the instruction. The stained cells were detected by flow cytometer (NovoCyte™ 2060R, ACEA Biosciences, China).

### RNA extraction, library construction, and sequencing.

After culturing for 30 days, total RNA was extracted from each group (containing three parallel samples) using RNAiso Plus reagent (Takara) following the instruction. A sequencing library was generated using the TruSeq RNA Sample Preparation Kit (Illumina). Then, mRNA was isolated and purified from the total RNA by magnetic beads with poly-T oligonucleotide attached. The divalent cations in Illumina’s proprietary fragmenting buffer played the dominant part in fragmentation at high temperatures. Random oligonucleotides combined with SuperScript II produced the first strand cDNA, followed by the generation of the second strand cDNA by DNA polymerase I and RNase H. Under the action of exonucleases and polymerases, the rest overhangs were transformed to blunt ends and then removed. After treating the 3′ ends of the DNA fragments by adenylation, the Illumina PE adapter oligonucleotides were bound together for hybridization. The AMPure XP system (Beckman Coulter) was used to purify the library fragments, so as to pick out 200 bp-long cDNA fragments. In a PCR reaction with 15 cycles, the DNA fragments attached with adapter molecules at both ends were selectively enriched by Illumina PCR Primer Cocktail. AMPure XP system was used to purify the products before a high sensitivity DNA assay was conducted to quantify the products on a Bioanalyzer 2,100 system (Agilent). Finally, a HiSeq platform (Illumina) was used to sequence the library.

### Transcriptome assembly and gene annotation

After sample sequencing, image files were obtained, which were then converted into FASTQ format data (Raw Data). The sequencing data were screened by the Cutadapt (v1.15) software to obtain sequences with good quality (Clean Data). HISAT2 (http://ccb.jhu.edu/software/hisat2/index.shtml) was used to the filtered reads to input into the reference genome, and the comparative region distribution of mapped reading fragments was calculated. Firstly, each gene’s count values read were compared as the initial expression of the gene by using HTSeq (0.9.1) statistics, and then the expression was standardized by FPKM. Secondly, DESeq (1.30.0) was used to analyze the DEGs under the screened conditions of expression difference multiple |log2FoldChange| > 1 as well as significant *p*-value < 0.05. At the same time, the two-way cluster analysis of all the genes differentially expressed in samples was conducted using R language Pheatmap (1.0.8) software package. The distance was calculated by the Euclidean method and the heat map was obtained by using the complete linkage method based on the expression of the same gene in various samples and distinct genes in the same sample. Next, we counted the number of genes differentially enriched in each Term after all genes were input into Terms of the Gene Ontology (GO) database. Terms significantly enriched by DEGs were summarized by the hypergeometric distribution method on the basis of the whole genome, in order to reveal possible functions of these genes in samples. We also numbered genes differentially expressed in KEGG pathway at distinct levels, and the metabolic and signaling pathways involving DEGs were identified. Moreover, GSEA of all detected genes was conducted by GSEA software (version 3.0), which is on the basis of gene sets to analyze different biological functions between two groups.

### Gene expression data sets

The GSE26168 dataset was obtained from the public Gene Expression Omnibus (GEO) database (http://www.ncbi.nlm.nih.gov/geo) and normalized using Robust Multichip Average (RMA). The student *t*-test was performed to compare differences between the experimental group and the control group. A *p*-value of 0.05 indicated statistical significance. IBM SPSS Statistics 20.0 was used for statistical analysis.

### Cell proliferation assay

After cell culture in normal or high glucose medium (αMEM with 5.6 mM or 35 mM glucose) for 30 days, BMSCs were seeded at a density of 5 × 10^3^ cells/well in a 96-well plate and induced with different concentrations of oleuropein for 1, 3, 7 days respectively. BMSCs proliferation was assessed using a cell counting kit-8 (CCK-8, Sangon, China). The absorbance at the wavelength of 450 nm was measured with a universal microplate spectrophotometer (Thermo LabSystems, Beverly, MA, United States).

### Alkaline phosphatase assay

After cultured in normal or high glucose medium (αMEM with 5.6 mM or 35 mM glucose) for 30 days, BMSCs were seeded in a 48-well dish at a density of 2 × 10^4^ cells/well. After adhered, the cells were cultured in osteogenic medium (Cyagen, China) with normal glucose (5.6 mM, LG group) and high glucose (35 mM, HG group), which were then induced with different concentrations of oleuropein (0, 3.125, 6.25, 12.5, 25, and 50 μM) (MCE, China) for another 7 days. ALP staining was performed in accordance with the instructions of BCIP/NBT Alkaline Phosphatase Kit (Beyotime, China), and the semi-quantitative analysis of ALP activity was operated based on the protocol of Alkaline Phosphatase Assay Kit (Nanjing Jiancheng, China).

### Quantitative real-time PCR

After 7 days of osteogenesis induction, RNAiso Plus (Takaro, Japan) was used to extract total RNA of different groups according to the manufacturer’s instructions. Applying PrimeScript™ RT Master Mix kit (Perfect Real Time) (Takara, Japan) to reverse transcription. qPCR was performed *via* the TB Green Premix Ex Taq™ kit (Tli RNaseH Plus) (Takara) in LightCycler@ 96 Instrument (Roche). β-tubulin was standardized as the internal reference. Primer sequences used in this study were listed in Table 1.Each sample was assayed in triplicate. Data analysis adopted 2^−ΔΔCT^ calculation method.

**TABLE 1:** Primer sequences used in this study.

Primer	Primer sequences
Runx2	F: GAC​TGT​GGT​TAC​CGT​CAT​GGC
	R: ACT​TGG​TTT​TTC​ATA​ACA​GCG​GA
Osterix	F: CCT​TCC​CTC​ACT​CAT​TTC​CTG​G
	R: TGT​TGC​CTG​GAC​CTG​GTG​AGA​T
OCN	F: GCA​GGA​GGG​CAA​TAA​GGT​AGT
	R: GCG​GTC​TTC​AAG​CCA​TAC​TG
β-tubulin	F: CTG​CTC​ATC​AGC​AAG​ATC​AGA​G
	R: GCA​TTA​TAG​GGC​TCC​ACC​ACA​G
Wnt10b	F: GCG​GGT​CTC​CTG​TTC​TTG​G
	R: CCG​GGA​AGT​TTA​AGG​CCC​AG

### Data analysis

The Student *t*-test was performed to compare differences between two groups. One-way analysis of variance was used for comparison among multiple groups, followed by a stepwise comparison between groups. α = 0.05, *p* < 0.05 considered as statistically significant. In the chart, statistical differences are indicated by “*ˮ, “*” means *p* < 0.05, “**”means *p* < 0.01, and “***”means *p* < 0.001. IBM SPSS Statistics 20.0 was used for statistical analysis.

## Results

### Flow cytometry results

**FIGURE 1:**
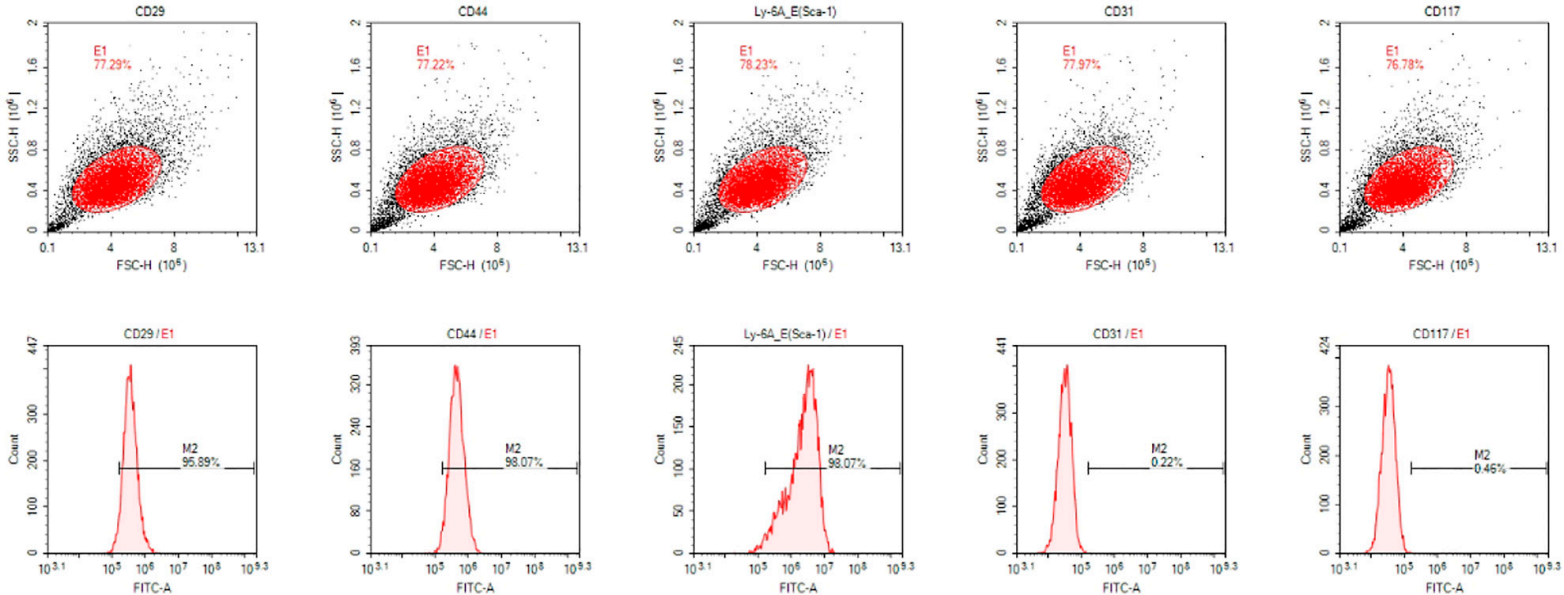
The results of flow cytometry.

The results of flow cytometry were shown in Figure 1. The positive expression rates of surface markers CD29, CD44, and Sca-1 of mesenchymal stem cells were 95.89%, 98.07%, and 98.07% respectively, while the positive expression rates of CD31 and CD117 were only 0.22% and 0.46% respectivley. These results indicated that the cells we extracted were identified as stem cells for their highly expressed surface markers of mesenchymal stem cells.

### Sequencing, *de novo* assembly, and functional annotation

**FIGURE 2:**
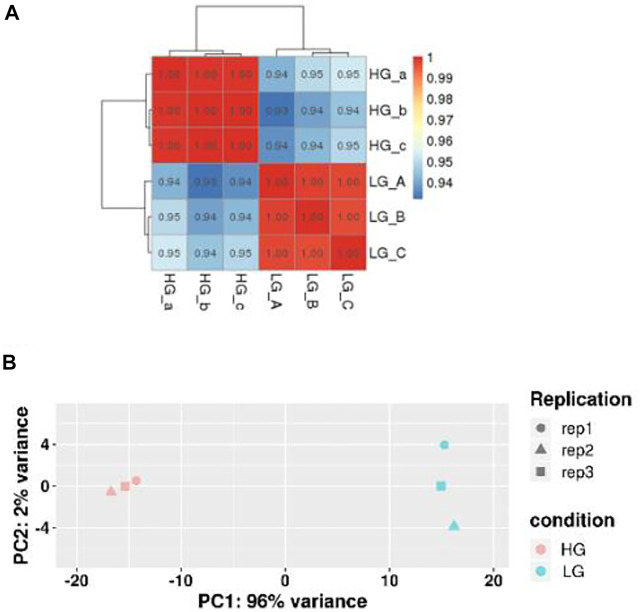
Correlation of gene expression levels between samples. **(A)** Sample Correlation Test. The closer the correlation coefficient was to one, the more similar the expression patterns between samples were. **(B)** Principal Components Analysis. The closer the samples, the higher the degree of similarity.

RNA-seq analysis of three parallel samples was carried out (three control samples and three experimental samples). We collected equal quantities of total RNA from the three replicates of the control and experimental groups to minimize the errors caused by individual differences. The assembled transcriptomes of BMSCs had a good quality (Table 2). Pearson correlation coefficient was employed to represent the correlation of gene expression levels between samples (Figure 2A). The expression patterns of the parallel samples from two groups were quite similar. Principal Components Analysis (PCA) also showed very similar results between samples (Figure 2B).

**TABLE 2:** RNASeq Map statistics.

Sample	Clean reads	Total mapped	Multiple mapped	Uniquely mapped
HG_a	51040814	49525914 (97.03%)	1971785 (3.98%)	47554129 (96.02%)
HG_b	40477830	39400235 (97.34%)	1562007 (3.96%)	37838228 (96.04%)
HG_c	43331230	42167765 (97.31%)	1672053 (3.97%)	40495712 (96.03%)
LG_A	4365278	42250986 (96.78%)	1591466 (3.77%)	40659520 (96.23%)
LG_B	53030162	51361031 (96.85%)	2061740 (4.01%)	49299291 (95.99%)
LG_C	42047404	40707117 (96.81%)	1616699 (3.97%)	39090418 (96.03%)

### Differentially expressed genes

**FIGURE 3:**
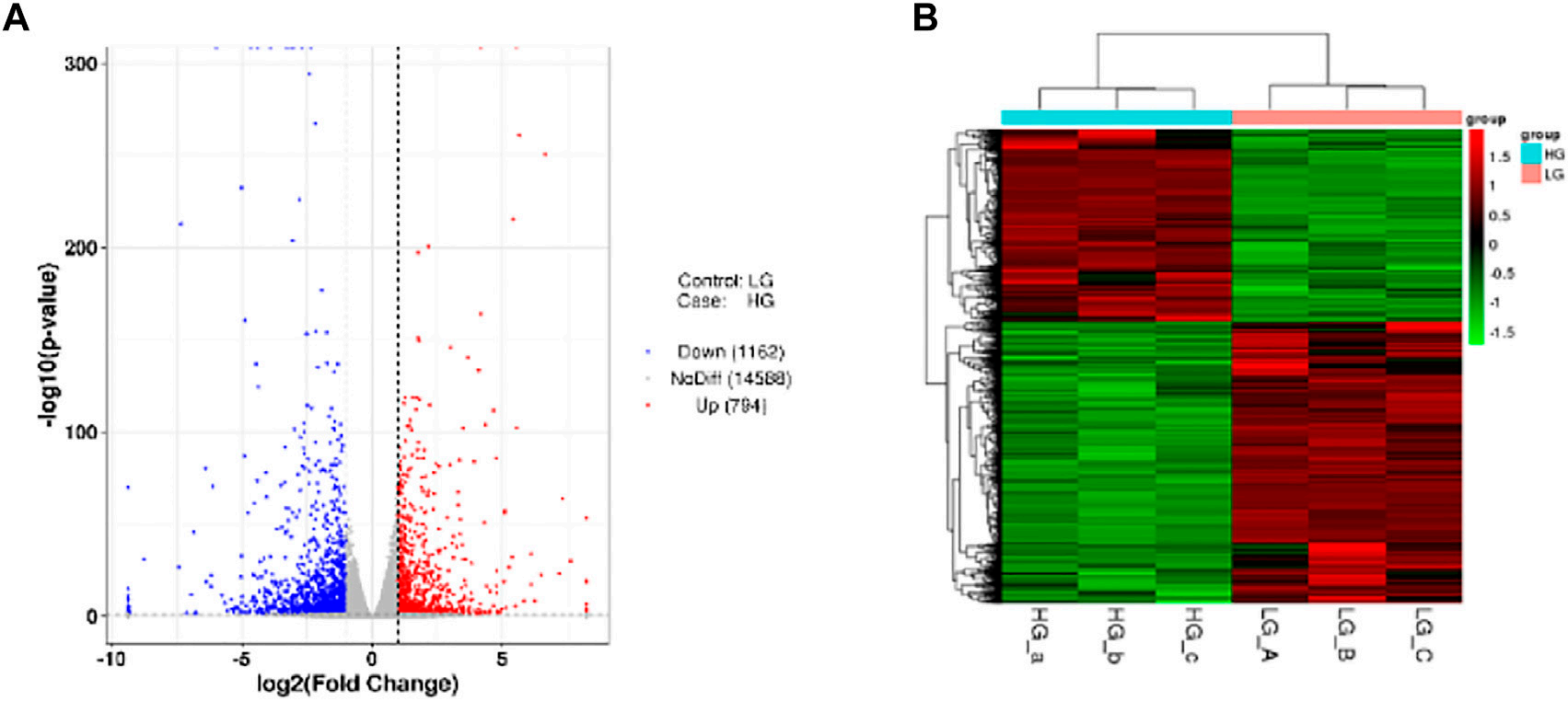
DEGs among the two groups. **(A)** The Volcano plot. The two vertical dashed lines mean 2 times the expression difference threshold. The dashed horizontal line stand for the threshold value of *p*-value = 0.05. The red dots are the upregulated genes HG group vs. LG group, while the blue dots are the downregulated genes, and the gray dots stand for the genes that are not significantly differentially expressed. **(B)** Cluster of differentially expressed genes. Red and green colors represent highly expressed genes and lowly expressed genes, respectively.

In order to study the gene expression in a high glucose environment, DESeq was used for differential analysis of genes expression. DEGs were screened under the conditions of |log2FoldChange| > 1 with significant *p*-value <0.05 of all DEGs, we identified that 794 genes were upregulated while 1,162 were downregulated when BMSCs were exposed to high glucose environment (Figure 3A). The cluster of differentially expressed genes was shown in Figure 3B.

### Functional analysis of DEGs

**FIGURE 4:**
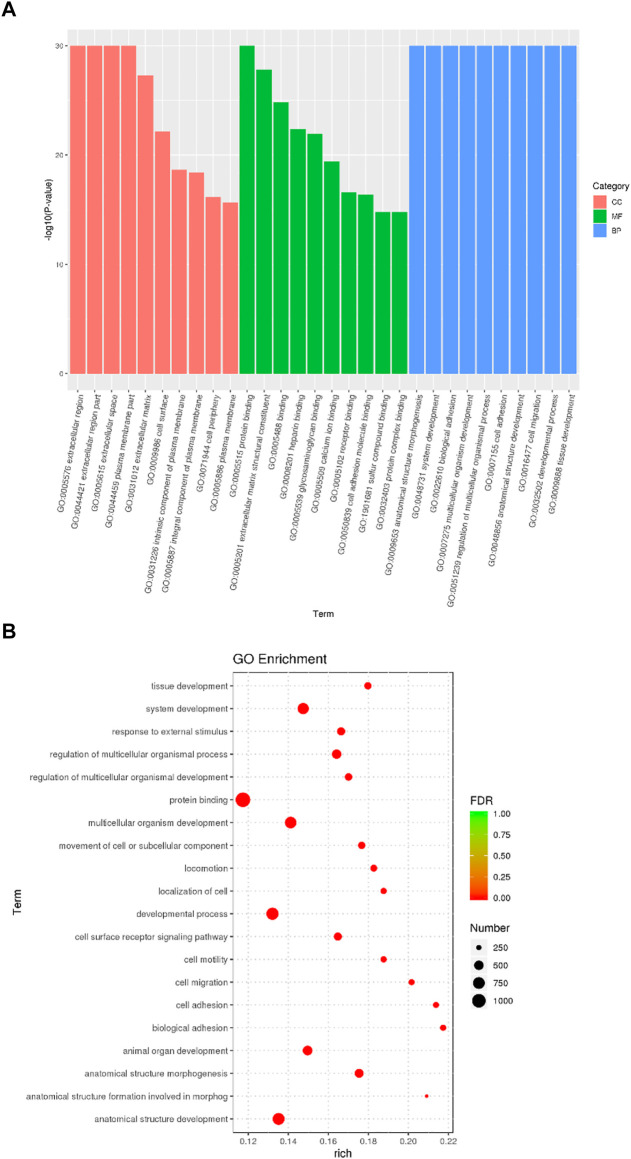
GO enrichment analysis of DEGs from BMSCs in high glucose microenvironment at 35 mM concentration for 30 days. **(A)** GO enrichment analysis histogram. **(B)** GO enrichment analysis bubble map.

**FIGURE 5:**
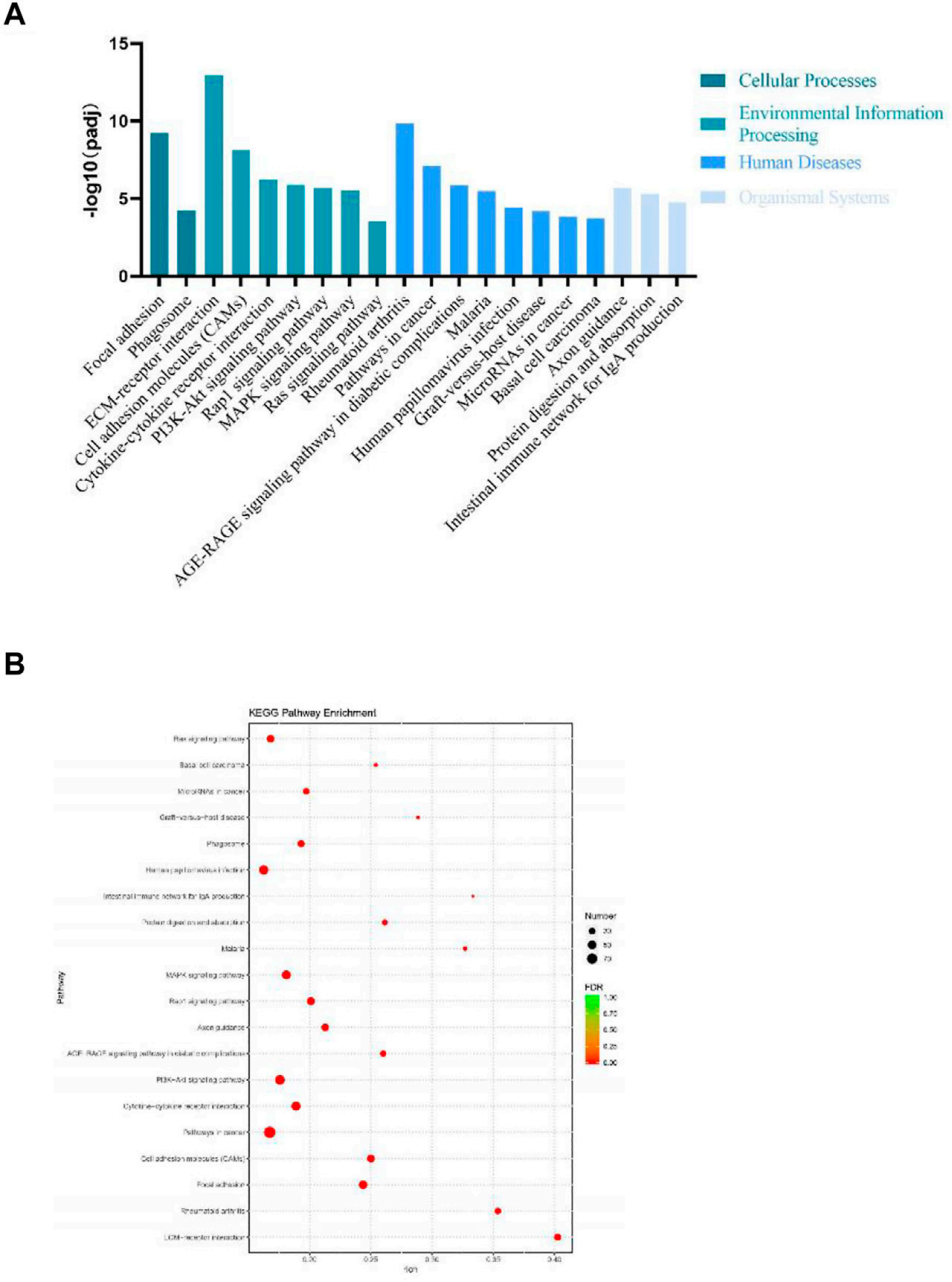
KEGG enrichment analysis of DEGs from BMSCs in high glucose microenvironment at 35 mM concentration for 30 days. **(A)** KEGG pathway enrichment analysis histogram. **(B)** KEGG enrichment analysis bubble map.

DEGs significantly enriched to GO terms or metabolic pathways were identified by the functional GO and KEGG enrichment analysis. As can be seen from the GO database, DEGs mainly included the following potential functions, namely, cellular component (CC), molecular function (MF) and biological process (BP). In each GO category, the top 10 GO term items with the smallest *p*-value have the most significant enrichment (Figure 4A). According to the GO enrichment results, the degree of enrichment was measured by the values of rich factor, FDR and the number of genes enriched to this GO Term. The top 20 GO Term items with the least FDR value have the most significant enrichments (Figure 4B). It is worth noting that the potential function functions of DEGs related to cell activities such as motility, migration and adhesion, as well as to the development of system, multicellular organism, anatomical structure and tissue. All the annotated KEGG pathways were grouped into four major types: cellular processes, environmental information, human diseases and organismal systems. The top 20 GO pathways with the smallest *p* value and enriched by most genes are shown in Figure 5A. Interestingly, we found our results verified some ideas in the field of diabetic suppression of bone formation. Just as Figure 5A illustrated, high glucose microenvironment influences biological process of stem cells such as migration, adhesion, which may be one of the main reasons why the diabetic condition proved to be detrimental for osteogenesis (Gilbert, 2013; Chen et al., 2021). Figure 5B shows the top 20 KEGG Term items having the most significant enrichment, including PI3K-Akt, Rap1, MAPK, Ras, and TGF-beta signaling pathways. PI3K-AKT pathways and MAPK pathways are both classical pathways that related to osteogenesis. Also, PI3K-AKT, Ras, and TGF-beta signaling pathways implied inflammation or reactive oxygen species (ROS) would be crucial to diabetic bone formation (Sanchez-de-Diego et al., 2019; Yuchen et al., 2020; Dawei et al., 2021; Zhang et al., 2021). Interestingly, two signaling pathways were found to be related to diabetes mellitus, i.e., AGE-RAGE pathway in diabetic complications and Type 1 diabetes mellitus. Activation of advanced glycation end products (AGEs) receptor by the high glucose enhanced the expression of inflammatory cytokines and contributed to chronic inflammation (Napoli et al., 2017).

### GSEA findings

**FIGURE 6:**
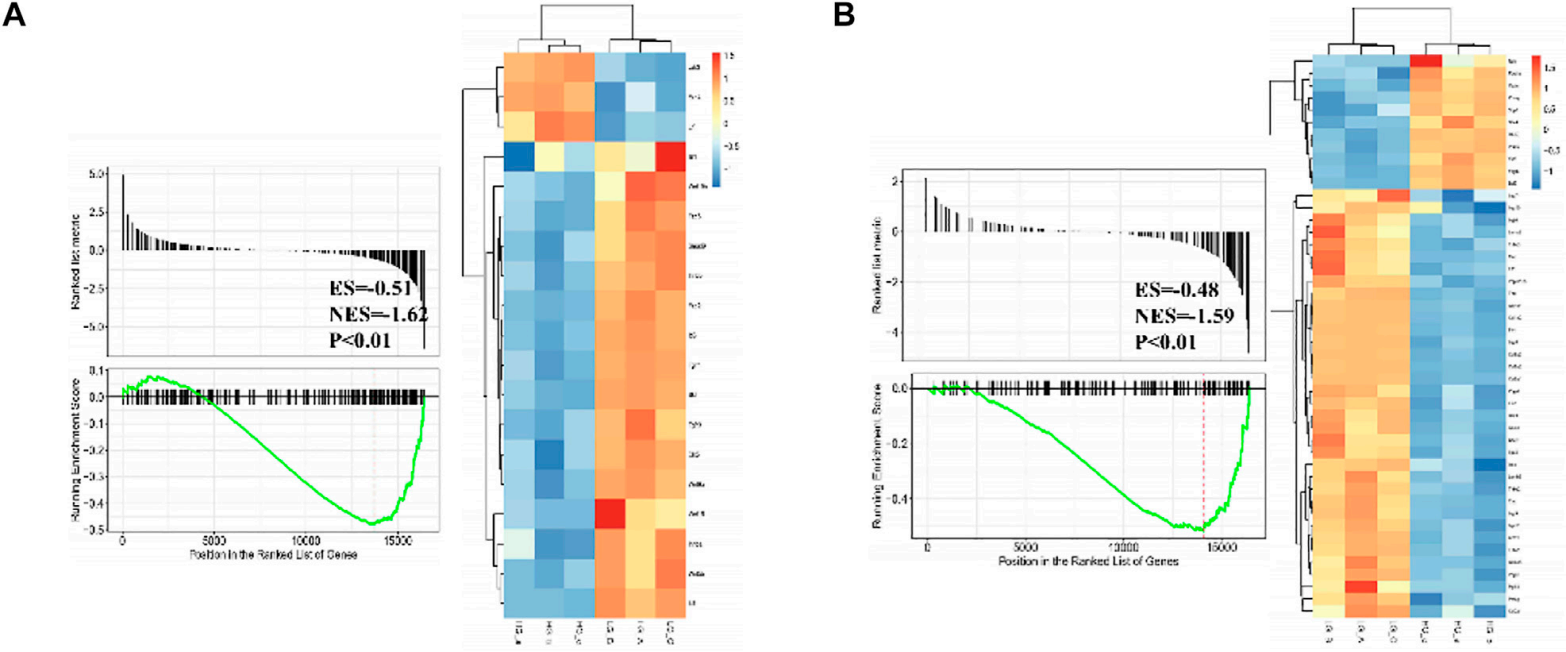
GSEA plot showing most enriched gene sets of all detected genes from BMSCs in high glucose microenvironment at 35 mM concentration for 30 days.

Differentially expressed gene sets between normal glucose and high glucose microenvironment were identified using with FDR < 0.25 and *p*-value < 0.001. The top two gene sets that were negatively related to high glucose condition were enriched in focal adhesion and signaling pathways regulating pluripotency of stem cells. DEGs related to these two gene sets were shown in Figure 6.

### Wnt10b was significantly downregulated in DM

**FIGURE 7:**
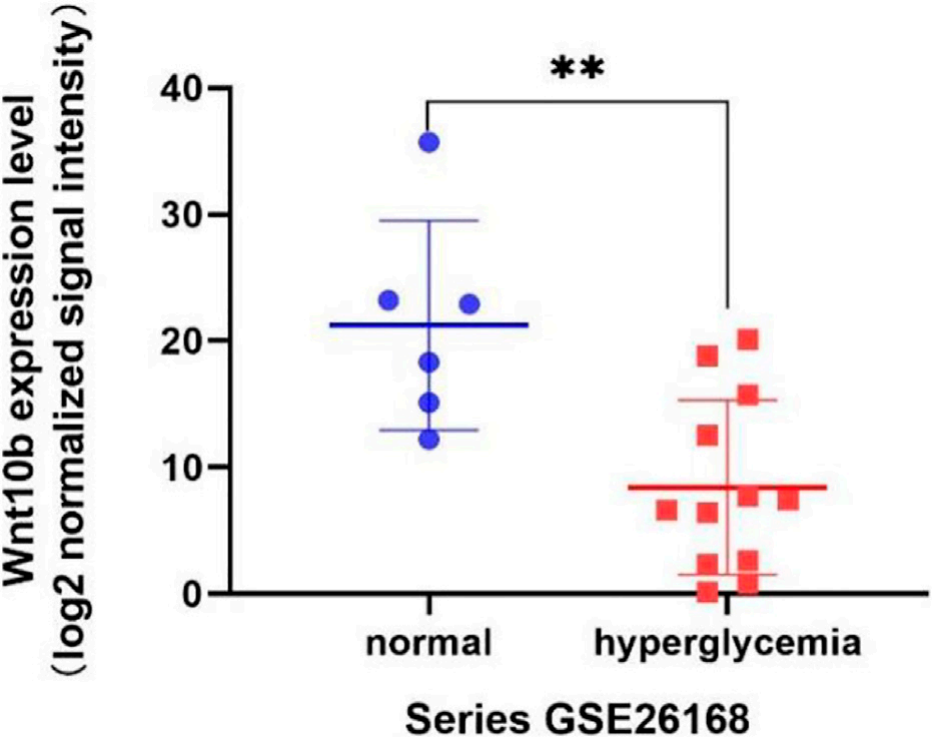
Wnt10b expression level from the GEO data sets (GSE26168).

According to the result of GSEA, genes associated with pluripotency regulation of stem cells were significantly down-regulated under a high glucose microenvironment, including Wnt10b. Through searching the public Gene Expression Omnibus (GEO) database, a previous study (GSE26168) analyzed the blood samples of male pre-diabetes and Type 2 Diabetes mellitus (T2DM) patients after an overnight fasting (Karolina et al., 2011). Then we reanalyzed the GSE26168 dataset and consistently found the down-regulated expression of Wnt10b in blood samples of people with impaired fasting glucose and DM (Figure 7).

### Oleuropein reversed the osteogenic suppression of BMSCs under the high glucose microenvironment *via* Wnt10b activation

**FIGURE 8:**
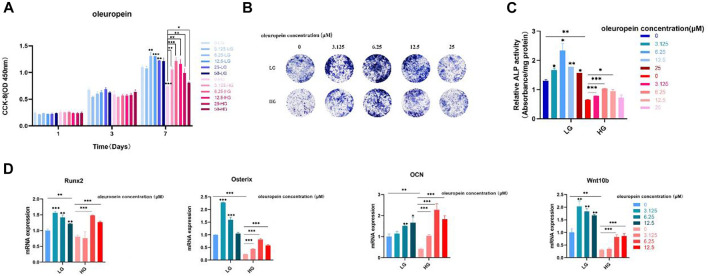
Oleuropein reverses the osteogenic suppression of BMSCs under the high glucose microenvironment. **(A)** CCK-8 analysis. **(B)** ALP staining. **(C)** Semi-quantitative analysis of ALP. **(D)** PCR analysis.

To further verity the function of Wnt10b in osteogenic activity of BMSCs, cells cultured with high-glucose medium were dealt with oleuropein, which proved to be an activator of Wnt10b (Park et al., 2011; Santiago-Mora et al., 2011; Kuem et al., 2014; Tong et al., 2015; Ahamad et al., 2019). The results of CCK-8 showed that the proliferation of BMSCs was suppressed under the high glucose microenvironment and 3.125 μM–25 μM oleuropein induction could promote the cell proliferation effectively (Figure 8A). ALP staining demonstrated that high glucose microenvironment results in significant osteo-inhibition, which was reversed by 3.125 μM–12.5 μM oleuropein induction (Figure 8B). Additionally, semi-quantitative analysis of ALP also confirmed these findings (Figure 8C). According to CCK-8 and ALP experiments’ results, we chosen 6.25 μM–12.5 μM concentration of oleuropein that appeared better abilities of promoting cell proliferation and osteogenesis for further exploring. qRT-PCR results have shown the down-regulated mRNA levels of osteogenesis-associated genes, including Runx2, Osterix and OCN in BMSCs cultured with high glucose medium for 7 days, and their levels were up-regulated by 6.25 μM–12.5 μM oleuropein induction, as well as the mRNA level of Wnt10b (Figure 8D).

## Discussions

Elevated blood glucose level is the major characteristic of DM, leading to the high glucose microenvironment in which various tissues are affected and then a series of complications occur. Osseous tissues are composed of multiple types of cells, including osteoblasts, osteoclast, osteocytes and BMSCs that suffer from the change of bone microenvironment (Shahen et al., 2020). BMSCs are featured by the differentiation potential to multi-lineage cells, including osteoblasts, and they play a critical in bone metabolism. Transcriptional control is necessary for differentiation of BMSCs. However, the transcriptional change of BMSCs under high glucose microenvironment is largely unknown. In this paper, mouse BMSCs were exposed to a 35 mM-glucose microenvironment for continuous 30 days to simulate the uncontrolled chronic high glucose environment (Li et al., 2019; Chen et al., 2022). Through the whole transcriptional sequencing, a total of 794 up-regulated genes and 1,162 down-regulated genes were preliminarily identified.

BMSCs osteogenesis is driven by various signaling pathways, such as the Hedgehog pathway (Tu et al., 2012), the Notch pathway (Canalis et al., 2016), the Wnt/β-catenin pathway (Wang et al., 2012), etc. The influence of signaling pathways on the high glucose microenvironment has been rarely reported. Jyoti Shrestha Takanche et al. (2020) suggested that gomisin A could regulate osteoblast differentiation under the oxidative stress condition induced by high glucose (25 mM D-glucose companied with 5 mU/ml glucose oxidase) through upregulating HO-1 and maintaining mitochondrial homeostasis. Xiaozhou Ying et al. (2015) confirmed that high glucose (30 mM) suppresses osteogenic differentiation of human BMSCs, but the activated PI3K/Akt signaling pathway by silibinin induction can reverse the osteogenic dysfunctions. Zhuling Jiang et al. (2019) reported that high glucose (4,500 mg/l glucose) results in osteoblastic dysfunctions, which, however, can be reversed by the activation of Shh signaling pathway. According to the functional analysis of EDGs, we targeted a few signaling pathways that deserved to be further tapped, including the PI3K-Akt, Rap1, MAPK, Ras, TGF-β and AGE-RAGE signaling pathways.

From the GSEA result, two significant gene sets related to high glucose exposure were discovered, including the Focal adhesion and Signaling pathways regulating pluripotency of stem cells. Multitude of genes associated to pluripotency regulation of stem cells significantly witnessed a significant drop under the high glucose microenvironment, including Wnt10b. To further vertify the connection between Wnt10b expression and DM, we searched in public Gene Expression Omnibus (GEO) database and found a previous study (GSE26168) that analyze the blood samples from male pre-diabetes and T2DM patients (Karolina et al., 2011). Consistently, we revealed that there is also a decrease in the expression of Wnt10b in the blood samples after reanalysis. Several studies have proven that the expression of Wnt10b is inhibited in DM patients (Abiola et al., 2009; Janssen et al., 2020). Wendy et al. suggested that mice with acitvated Wnt10b present decreased bone loss, obesity inhibition, increasing insulin sensitivity and glucose tolerance (Wright et al., 2007). Besides, Wnt10b plays a vital role in driving osteogenesis and inhibiting adipogenesis (Collins et al., 2017). Bennett et al. (2002) Bennett et al. (2005) have validated that Wnt10b^−/−^ mice presents a significant decrease in osteoblasts, while Wnt10b promoter drives bone formation. Previous researches also demonstrated that Wnt10b is a crucial regulator of the mesenchymal progenitor fate, mainly through changing bone formation rather than bone resorption (Wend et al., 2012).

To further verify the function of Wnt10b in high glucose-induced osteogenesis inhibition, BMSCs were induced with varying concentrations of oleuropein, which has been validated as Wnt10b activator (Park et al., 2011; Kuem et al., 2014). Oleuropein is acquired from leaves and fruit oil, which is the most active phenolic compound in olive oil and regarded as one of the most beneficial compound of it (Drira et al., 2011; Ahamad et al., 2019). It has been strongly validated that oleuropein is functional in anti-oxidant, anti-inflammation, anti-diabetic, cardioprotective, anticancer and hepatoprotective actions (Ahamad et al., 2019). Nowadays, the biological function of oleurpein in bone regeneration has been well concerned (Puel et al., 2006; Santiago-Mora et al., 2011). A previous study discovered that oleuropein can regulate stem cells differentiation, prevent bone loss or osteoporosis (Santiago-Mora et al., 2011). In our research, we validated the outstanding effect of oleuropein in promoting the proliferation and differentiation of BMSCs under a high glucose microenvironment through CCK-8 and ALP analysis respectively. qRT-PCR also proved that oleuropein up-regulated Wnt10b, as well as the osteogenesis-related genes Runx2、Osterix and OCN.

In conclusion, the results of RNA-seq showed that the high glucose microenvironment could significantly influence the cell adhesion and pluripotency regulation of BMSCs. Wnt10b has been discovered as a potential biomarker and therapeutic target to reverse high glucose-induced osteogenic suppression, and oleuropein might be an effective drug in the treatment of diabetic osteopathy. Other potential transcriptional factors and signal molecules involved in the osteogenesis of BMSCs should be further investigated in the future, and their molecular mechanisms, thus more clearly clarifying the transcriptional regulation of the dynamic changes in osteogenesis under the high glucose microenvironment. Our results may provide new insight into diabetic osteopathy.

## Data Availability

The data presented in the study are deposited in the NCBI repository, accession number PRJNA861406.

## Fuding

We acknowledge support by the Natural Science Foundation of China (No. 82071081), Special Project of Clinical Research in Health Industry of Shanghai Municipal Health Commission (Excellence Program) (No.20224Z0010) and Clinical Research and Key Support Project of Shanghai ShenKang Hospital Development Center (No. SHDC2020CR5015).
